# Role of Palatine Tonsil and Epipharyngeal Lymphoid Tissue in the Development of Glomerular Active Lesions (*Glomerular vasculitis*) in Immunoglobulin A Nephropathy

**DOI:** 10.3390/ijms23020727

**Published:** 2022-01-10

**Authors:** Osamu Hotta, Norio Ieiri, Masaaki Nagai, Ayaki Tanaka, Yasuaki Harabuchi

**Affiliations:** 1 Division of Internal Medicine, Hotta Osamu Clinic (HOC), Sendai 984-0013, Miyagi, Japan; ieiri@hoc.ne.jp; 2 Division of Nephrology, Narita Memorial Hospital, Toyohashi 441-8029, Aichi, Japan; nagai5823@meiyokai.or.jp; 3 Tanaka ENT Clinic, Osaka 553-0006, Japan; tuvajp@gmail.com; 4 Department of Otolaryngology-Head and Neck Surgery, Asahikawa Medical University, Asahikawa 078-8510, Japan; hyasu@asahikawa-med.ac.jp

**Keywords:** glomerular vasculitis, IgA nephropathy, epipharynx–kidney axis, chronic epipharyngitis, chronic tonsillitis

## Abstract

Hematuria is an essential symptom of immunoglobulin A nephropathy (IgAN). Although the etiology of hematuria in IgAN has not been fully elucidated, it is thought that the rupture of the glomerular basement membranes caused by intra-capillary leukocyte influx, so-called glomerular vasculitis, is the pathological condition responsible for severe hematuria. Glomerular vasculitis are active lesions that exist in the glomeruli of acute phase IgAN and it is important because it is suspected to make the transition to segmental glomerular sclerosis (SGS) as a repair scar lesion in the chronic phase, and the progression of SGS would eventually lead to glomerular obsolescence. Worsening of hematuria concomitant with acute pharyngitis is common in patients with IgAN; therefore, elucidating the relationship between the immune system of Waldeyer’s ring, including the palatine tonsil and epipharyngeal lymphoid tissue, and the *glomerular vasculitis* may lead to understanding the nature of IgAN. The epipharynx is an immunologically activated site even under normal conditions, and enhanced activation of innate immunity is likely to occur in response to airborne infection. Hyperactivation of innate immunity via upregulation of Toll-like receptors in the interfollicular area of the palatine tonsil and epipharyngeal lymphoid tissue, followed by enhanced fractalkine/CX3CR1 interactions, appears to play an important role in the development of *glomerular vasculitis* in IgAN. As latent but significant epipharyngitis is present in most patients with IgAN, it is plausible that acute upper respiratory infection may contribute as a trigger for the innate epipharyngeal immune system, which is already upregulated in a chronically inflamed environment. Given that epipharyngitis and its effects on IgAN are not fully understood, we propose that the so-called “epipharynx–kidney axis” may provide an important focus for future research.

## 1. Importance of Hematuria in Immunoglobulin A Nephropathy (IgAN)

**Figure 1:**
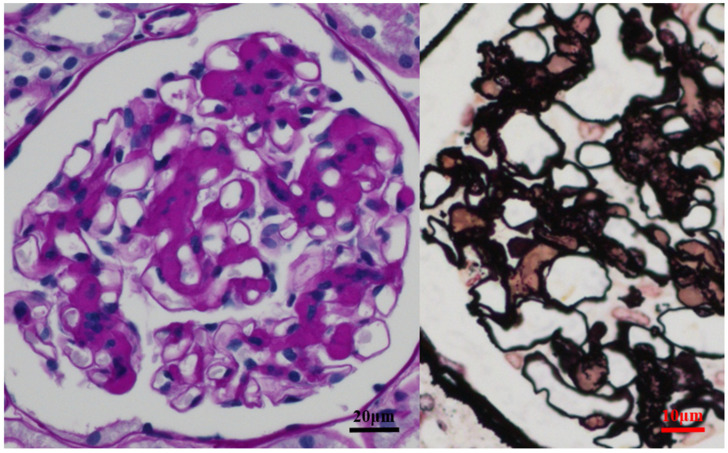
Mesangial expansion in immunoglobulin A nephropathy (IgAN) with massive IgA deposition. In cases with massive IgA deposition, pronounced hemispherical deposits are often observed (left PAS). Regardless of the extent of mesangium proliferation in response to IgA deposition, it is located in the extra-capillary area (brown) and did not occlude the glomerular vascular cavity (right, PAM).

Immunoglobulin A (IgA) deposition in the glomerular mesangium with mesangial cell proliferation is the histological hallmark of IgAN. However, more than 10% of normal individuals lacking urinary abnormalities have been reported to have mesangial IgA deposits [1]. Moreover, the degree of mesangial IgA deposits is not correlated with any clinical characteristics, including the magnitude of proteinuria and hematuria or the speed of progression of nephropathy [2] (Figure 1). Therefore, it is conceivable that mesangial IgA deposition is not a direct pathogenetic element but is among the necessary conditions responsible for the clinical manifestations of IgAN.

While most patients are asymptomatic, hematuria is an essential symptom of IgAN. Although the etiology of hematuria in IgAN has not been fully elucidated, it is believed that rupture of glomerular basement membranes caused by intra-capillary leukocyte influx is the pathological event responsible for aggravation of hematuria [3,4,5]. Such active lesions in glomeruli are also called “glomerular vasculitis” by some nephrologists as well as pathologists.

During the repair process of the ruptured glomerular basement membrane, all three types of glomerular cells, namely, mesangial cells, glomerular endothelial cells, and podocytes, dynamically adhere to each other and contribute to the formation of repair scar lesions [6,7].

**Figure 2:**
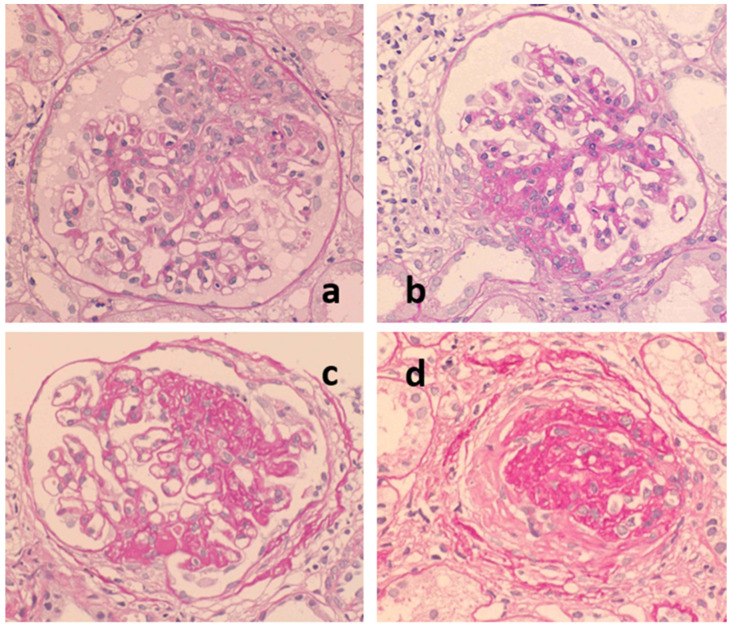
The glomerular sclerosing process following glomerular vasculitis. Following glomerular vasculitis (**a**), segmental glomerular sclerosis develops as a healing scar lesion (**b**). The accumulation and expansion of segmental glomerular sclerosis lesions (**c**) ultimately lead to glomerular obsolescence (**d**). PAS, original magnification ×400.

As the extracellular matrix used for tissue repair is produced primarily by mesangial cells, the mesangial region at the site where the *Glomerular vasculitis* develops expands and eventually forms a segmental sclerosis (SGS) (Figure 2). Therefore, *Glomerular vasculitis* preceding SGS lesions is important because the accumulation and expansion of SGS lesions are strongly associated with the progression of nephropathy. However, pathological evidence of *Glomerular vasculitis* is sometimes difficult to detect because the lesion is focal and segmental in nature, especially in the early disease phase, and because the number of glomeruli obtained by renal biopsy is limited [8]. Contrary to the lesion with glomerular vasculitis, glomerular lesions with mesangial expansion and IgA deposition are easily detectable because the lesions are diffuse and global.

Although the clinical significance of hematuria in IgAN has not been considered convincing as that of proteinuria, recent cohort studies have shown that remission of hematuria, spontaneously or after receiving immunosuppressive treatment, improves long-term renal survival in IgAN [9,10]. The link between hematuria remission and improved long-term renal prognosis appears reasonable if hematuria remission represents the termination of glomerular vasculitis.

**Figure 3:**
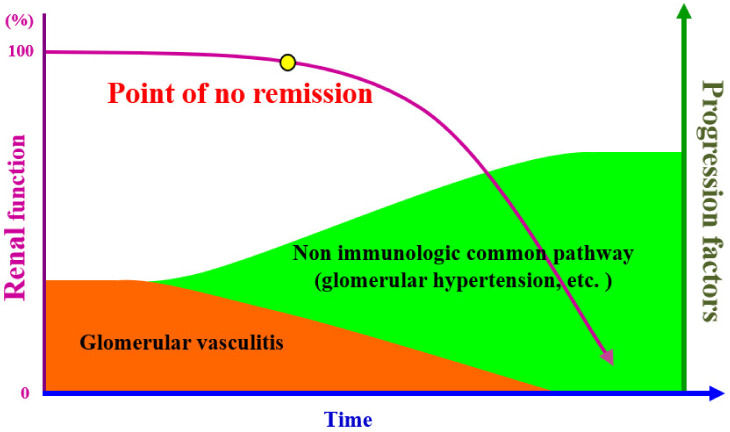
Progression factors in the time course of immunoglobulin A nephropathy (IgAN). *Glomerular vasculitis* is the main progression factor in the early stages of IgAN. Meanwhile, common pathways, such as glomerular hypertension, ischemia, and proteinuria-related tubulointerstitial injury, are the main contributing progression factors in the later stage of IgAN. *Glomerular vasculitis* can be extinguished by an effective therapeutic intervention, such as steroid pulse and tonsillectomy, but complete blocking of the common pathway is impossible for now. Therefore, there is a “point of no remission” in the time course to achieve remission or cure of IgAN.

Given that the core pathological condition of IgAN is “smoldering *glomerular vasculitis* with mesangial IgA deposition,” *Glomerular vasculitis* itself is the main progressive factor in the early stage of IgAN. Recently, crescent, a histological hall mark of glomerular vasculitis, has been considered to be a predictor of poor prognosis of IgAN [11]. Meanwhile, as nephropathy progresses and glomeruli with SGS lesions increases, the effects of other progressive factors, such as glomerular hyperfiltration/glomerular hypertension, tubulointerstitial injury, and kidney ischemia, which are the “common pathways”, become predominant (Figure 3). It is important to realize the essential difference in two types of glomerular lesions in the management of patients with IgAN: “While *Glomerular vasculitis* is a reversible condition [12], SGS is an irreversible condition”. Therefore, the “point of no remission” in the long course of IgAN should be regarded as an essential point. If effective therapy, such as tonsillectomy combined with steroid pulse (TSP) therapy, is undertaken before the “point of no remission,” the likelihood of clinical remission is high, while if treatment is initiated after this point, clinical remission may no longer occur [13], and consequently, slowing the progression of nephropathy would be the alternative goal of the treatment.

In this review article, we discuss the role of tonsillar and nasopharyngeal mucosal immunity in the development of glomerular vasculitis.

## 2. Role of Palatine Tonsil in IgAN

It is widely recognized that mucosal immunity plays an important role in the pathogenesis of IgAN. Hematuria in patients with IgAN often worsens concomitantly with acute pharyngitis. Although the pathological factors that worsen hematuria have not been fully elucidated, activation of the innate immune system of the upper respiratory tract, known as Waldeyer’s ring, is believed to play an important role. Palatine tonsils and nasopharyngeal (epipharyngeal) lymphoid tissue are the main lymphatic organs of Waldeyer’s ring, which may be involved in the pathogenesis of *Glomerular vasculitis* in IgAN concomitant with acute pharyngitis.

### 2.1. Characteristics of the Palatine Tonsil

The palatine tonsils, a pair of left and right lymphatic tissues located in the oropharynx, are composed of B cell-dominant lymphocytes and a few myeloid cells; however, unlike normal peripheral lymph nodes, there are no afferent lymphatic vessels. Therefore, palatine tonsils are believed to function as induction sites in the oral mucosal immune system.

**Figure 4:**
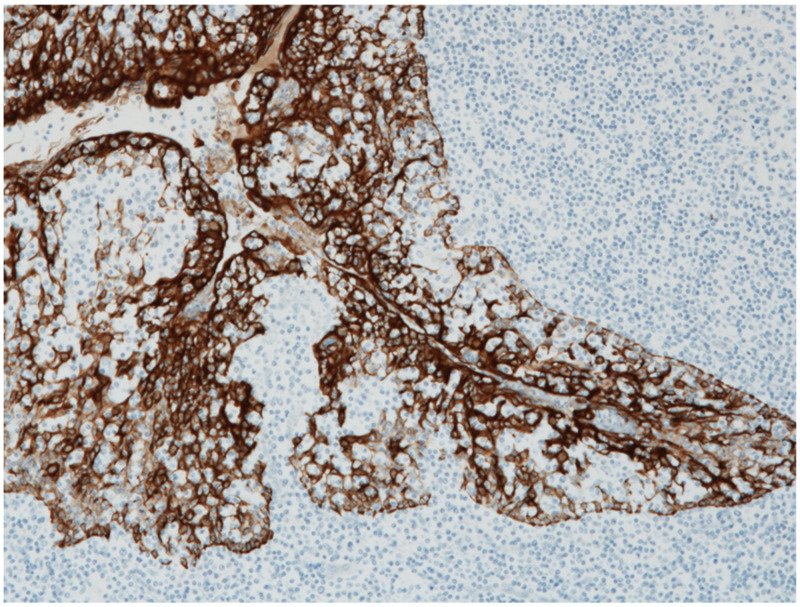
Lymphoepithelial symbiosis in palatine tonsils. The tonsillar crypt epithelium and tonsil parenchyma are mixed, which is called “lymphoepithelial symbiosis.” Reticular epithelial cells (cytokeratin positive brown cells)-fold lymphocytes (blue). Immunoperoxidase staining with anti-cytokeratin antibody (brown), original magnification ×100.

The surface of the palatine tonsils is covered with non-keratinizing squamous epithelium, which branches deeply into the tonsils to form crypts. The blind end of the crypt, where the crypt epithelium and tonsil parenchyma are mixed, is called lymphoepithelial symbiosis, and this structure is a characteristic of the palatine tonsils (Figure 4). Antigen-presenting cells, such as M cells (membranous epithelial cells) and dendritic cells as well as memory B cells, are distributed in the lymphoepithelial symbiosis site and are believed to be the starting point for antigen recognition in the tonsils.

**Figure 5:**
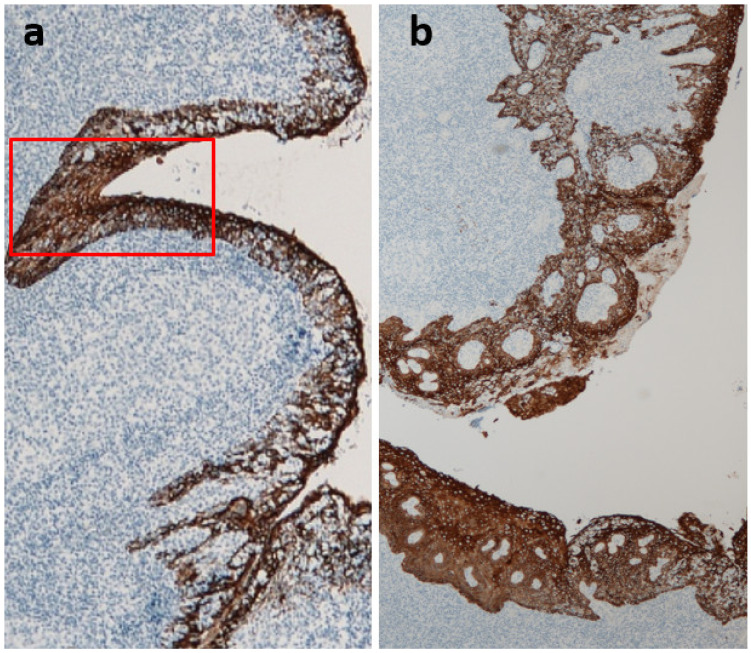
Reducing lymphoepithelial symbiosis and the progression of immunoglobulin A nephropathy (IgAN). The area of non-reticulated crypts (non-lymphoepithelial symbiosis areas) expands with the progression of IgAN. Representative cases: (**a**) CKD stage 2, disease period of 2 years, and (**b**) CKD stage 5, disease period of 30 years. The area of non-reticulated epithelia (red box) is restricted in the early stage of IgAN (**a**), whereas non-reticulation and squamous epithelialization are remarkable in ESKD IgAN (**b**). Stained with anti-cytokeratin antibody (brown), original magnification ×40.

The area of the non-reticulated crypt, that is, the non-lymphoepithelial symbiosis area, expands with the progression of glomerular damage in patients with IgAN, regardless of their age (Figure 5) [14,15].

The tonsil parenchyma is composed of B-cell-dominant lymphoid follicles and interfollicular areas. The interfollicular area is a T cell-dependent region, where T cells are mainly distributed and characteristically expanded in patients with IgAN [16,17].

### 2.2. Role of Tonsils in Mesangial IgA Deposition

Many recent clinical studies have clearly shown that TSP therapy is an effective treatment for patients with IgAN [18,19,20], and tonsillectomy is recommended as a treatment option based on evidence-based clinical guidelines for IgAN in Japan [21].

Therefore, IgAN has been regarded as a tonsil-related disease, more accurately, tonsil-induced autoimmune/inflammatory syndrome (TIAS), which is triggered by the breakdown of immune tolerance to resident bacteria in the tonsils [22].

Harabuchi et al. postulated that the tonsils of patients with IgAN have two important characteristics: (1) failure of immune tolerance against indigenous bacteria and (2) hyperimmune response against bacteria-originated DNA [22].

It is widely accepted that aberrantly glycosylated IgA1 is involved in the pathogenesis of mesangial IgA deposition. Recent studies indicate that overproduction of aberrantly glycosylated and polymeric IgA1 through BAFF-mediated and APRIL-mediated T cell-independent pathways in lymphoid follicles in tonsils may be a source of mesangial IgA deposition [23,24,25].

### 2.3. Role of Tonsils in Glomerular Vasculitis

To date, there is less knowledge of tonsillar immunity and its relation to *glomerular vasculitis* than aberrant IgA production in tonsils in relation to glomerular IgA deposition.

**Figure 6:**
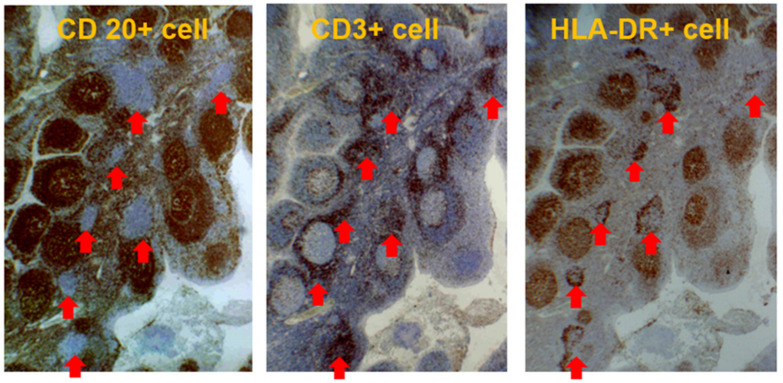
T cell nodules in palatine tonsil. T cell nodules (red arrows) consisting of CD3+CD20-HLA-DR+ cells present in the interfollicular area of the palatine tonsil (original magnification ×40).

The interfollicular area, which is expanded in the tonsils of patients with IgAN, is an important aspect of antigen-specific T cell activation [16]. Enlarged T cell nodules in the interfollicular areas of the tonsils have been reported to be characteristic of patients with IgAN [26,27]. The number of T cell nodules in the tonsils was correlated with the proportion of crescentic glomeruli, representing the severity of *Glomerular vasculitis* in renal biopsy tissues [15,17,27]. Furthermore, the T cells in the T cell nodules were positive for HLA-DR, an activation marker of T cells, indicating that the T cell nodules in the interfollicular area are composed of immunologically activated T cells (Figure 6). Furthermore, CD208+ dendritic cells collocated with T cell nodules, and the number of CD208+ dendritic cells in the tonsils was positively and linearly correlated with the proportion of crescentic glomeruli in renal biopsies [16]. CD208 is expressed almost exclusively by mature dendritic cells, and CD208 plays a key role in the processing and presentation of antigens during the immune response [28]. This co-localization of dendritic and T cells is efficient for dendritic cells that have processed information to activate and differentiate T cells.

CX3C chemokine receptor 1 (CX3CR1), the only CX3CL1 (fractalkine) receptor, is expressed in human NK cells, NKT cells, monocytes, CD8+ T cells, and γδ T cells [29]. Fractalkine, a single ligand of CX3CR1, is a unique molecule that acts as both an adhesion molecule and a chemokine [30]. Fractalkine is localized on the vascular endothelial surface and strongly attracts CX3CR1+ cells without the help of other adhesion molecules, while the soluble form acts as a chemokine that induces the migration of CX3CR1+ cells. Consequently, both membrane-bound and soluble forms of fractalkine act as strong chemoattractants for CX3CR1+ cells. Membrane-bound fractalkine can be induced in vascular endothelial cells by inflammatory cytokines such as tumor necrosis factor (TNF)-α, interferon-γ (IFN-γ), and interleukin-1 (IL-1) [31]. Moreover, the serum level of soluble fractalkine increases in patients with vasculitis, especially in the active stage [32].

Cox et al. studied patients with IgAN during episodes of macroscopic hematuria and observed an upregulated expression of the chemokine receptor CX3CR1 in circulating leukocytes [33]. Furthermore, Iwatani et al. reported that adoptive transfer of NK cell line cells or CD16+CD56+ cells derived from patients with IgAN into nude rats induced hematuria in the recipients. They also demonstrated that NK cells exert cytotoxic activity against human glomerular endothelial cells in a dose-dependent manner [34]. In addition, our preliminary study demonstrated a significant increase in the proportion of monocytes (macrophages), NK cells, CD8+ T cells, and γδ T cells (i.e., CX3CR1+ leukocytes) in the urine of patients with IgAN [35], indicating an enhanced fractalkine/CX3CR1 interaction in IgAN.

Notably, Otaka et al. demonstrated the upregulation of CX3CR1 in tonsillar CD8+ T cells in patients with IgAN and postulated that a hyperimmune response to microbial DNA enhanced the expression of CX3CR1 in CD8 + T cells in the interfollicular area of the palatine tonsils. This was followed by migration of CX3CR1+ cells to the kidneys through blood circulation, resulting in the development of hematuria caused by glomerular vascular injury [36].

**Figure 7:**
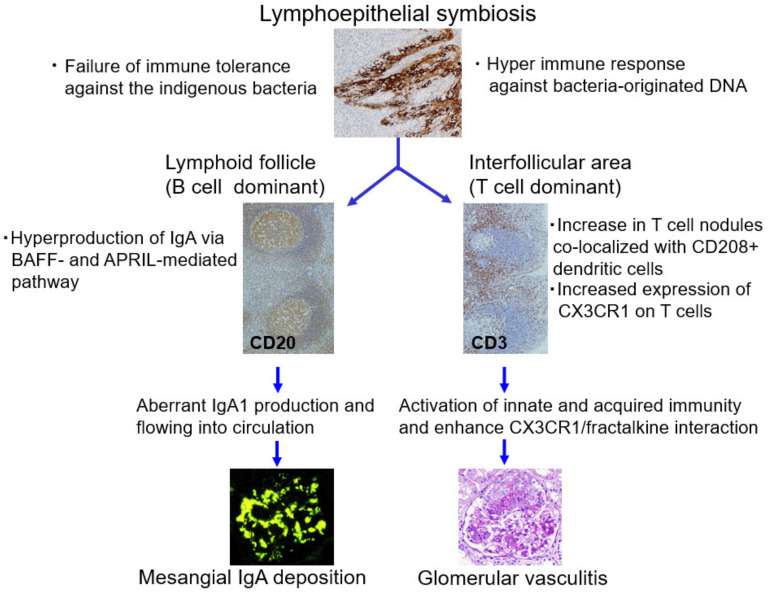
Role of palatine tonsils in the pathogenesis of immunoglobulin A nephropathy (IgAN). The palatine tonsil has a unique structure, namely, lymphoepithelial symbiosis, which functions as an induction site in the mucosal immune system. IgAN tonsils have characteristics such as failure of immune tolerance against indigenous bacteria and a hyperimmune response against bacteria-originated DNA. The overproduction of aberrantly glycosylated and polymeric IgA1 through BAFF and APRIL mediated T cell-independent pathways in lymphoid follicles in tonsils may be a source of mesangial IgA deposition. The increased number of T cell nodules co-localized with CD208+ dendritic cells in IgAN tonsils may be related to the enhanced expression of CX3CR1 in CD8+ cells and other types of lymphocytes with cytotoxic activity and the simultaneous upregulation of fractalkine/CX3CR1 interaction in distant glomeruli occurs, resulting in glomerular vasculitis.

Given that the increased number of T cell nodules co-localized with CD208+ dendritic cells in the tonsils of patients with IgAN is related to the enhanced expression of CX3CR1 in CD8+ cells and other types of lymphocytes with cytotoxic activity, the upregulation of fractalkine/CX3CR1 interaction in distant organs, such as the kidneys, should occur, resulting in glomerular vascular injury (Figure 7).

## 3. Role of the Epipharyngeal Lymphoid Tissue in IgAN

Macroscopic bouts, typically associated with acute pharyngeal infection, such as “synpharyngitic gross hematuria,” frequently occur in patients with IgAN. The triggering factor of gross hematuria in IgAN is presumably related to the aggravation of glomerular vasculitis, but this is poorly understood. Therefore, it is important to determine the factors that might promote macroscopic hematuria concurrent with acute pharyngitis. Some patients exhibit synpharyngitic gross hematuria even in the post-tonsillectomy state without any gastrointestinal symptoms. Moreover, residual hyperactivation of innate immunity has been demonstrated in tonsillectomized patients [37]. Thus, it is conceivable that an area other than the palatine tonsil or gut may be involved as a trigger for gross hematuria, that is, *Glomerular vasculitis* concomitant with acute pharyngitis.

Located at the back of the nasal cavity, the epipharynx is a unique tissue vulnerable to the effects of upper respiratory tract infections and air pollution. Furthermore, as a component of mucosa-associated lymphoid tissue (MALT), the epipharynx plays a role in the production of memory/effector T lymphocytes in response to exogenous antigens in inspired air, which contributes to host defense mucosal immunity as a source of antigen-specific IgA-producing B cells [38]. Inflammation of the epipharynx is not felt in the upper pharynx or in the back of the nose, but it presents as pain in the middle or lower pharynx. Epipharyngitis is the most frequent cause of throat pain [39]. The epipharynx is an immunologically activated site even under normal conditions, and enhanced activation of innate immunity is likely to occur in response to airborne infection. However, the condition and the role of the epipharynx in patients with IgAN have not been fully elucidated.

### 3.1. Anatomical, Physiological, and Immunological Characteristics of the Epipharynx

Because the epipharynx is the primary site of airway infection, it has a highly developed immunocompetent function to inhaled foreign proteins, such as those of bacteria and viruses.

Unlike the middle and lower pharynx, which are covered by stratified squamous epithelium, the surface of the epipharynx is lined with ciliated columnar epithelial cells. As a component of MALT, the epipharynx plays a role in both innate and acquired immunity.

During childhood, the pharyngeal tonsil (adenoid), which is part of the Waldeyer’s ring, is located on the roof of the epipharynx, posterior to the nasal cavity. Subsequently, the adenoids start to shrink before adolescence and usually disappear in adulthood. Although well-developed lymphoid follicles are restricted to the adenoid, the lymphoid tissue of the epipharynx is mainly composed of elements similar to those in the interfollicular region of the palatine tonsil.

**Figure 8:**
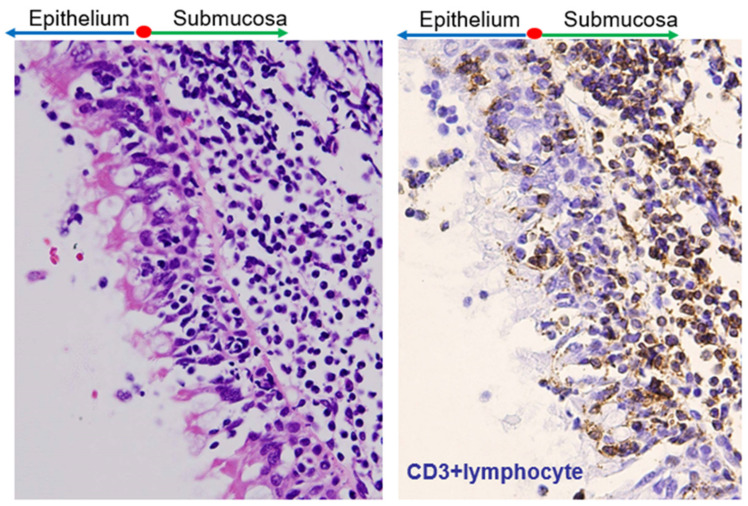
Surface of the epipharynx. There is an abundance of lymphocytes in the submucosal area (left, hematoxylin and eosin staining, original magnification ×100). Many lymphocytes are located within ciliated epithelial cells (right, indirect immunoperoxidase staining with anti-CD3 antibody, original magnification ×100).

Abundant lymphocytes exist mainly in the submucosal area, and it should be emphasized that many lymphocytes collocate among epipharyngeal epithelial cells [40] (Figure 8). The epipharynx is rich in dendritic cells, and some of them penetrate the tight epithelial junctions, which facilitate access to antigens [41]. Furthermore, membranous (M) cells are present in the epipharyngeal epithelium and serve as entry portals for antigens in the MALT of the upper respiratory tract [42], similar to those in the palatine tonsils and Peyer’s patches.

Epipharyngeal mononuclear cells predominantly contain B cells, approximately 5% macrophages, and 30–40% CD3+ T cells [40,43], while natural killer cells represent a minor cell population compared to the distribution in peripheral blood [44]. T cells are primarily the CD4+ subset (approximately 80%). Importantly, both T and B lymphocytes are highly activated, even in normal individuals. Notably, these characteristics of epipharyngeal lymphocytes are similar to those of palatine tonsillar lymphocytes. The activation of epipharyngeal CD4+ and CD8+ T cells is marked in patients with acute pharyngitis; notably, these cell activations are significantly more pronounced in patients with IgAN lacking acute pharyngitis symptoms than in normal individuals (Table 1).

### 3.2. Epipharyngeal Response to Airborne Infections

Upper respiratory tract infections are the most frequent type of infection that people experience in their lifetime. Epipharyngeal tissue copes with inhaled pathogens through multiple mechanisms: (1) removal of large particles and microorganisms through mucociliary clearance; (2) recognition of pathogens through pattern recognition receptors, primarily Toll-like receptors (TLRs); (3) secretion of proinflammatory cytokines and antimicrobial peptides; (4) activation of adaptive immunity, including the proliferation and differentiation of specific clones in the epipharyngeal lymphoepithelial tissue. In response to the invasion of pathogens into the epipharynx, neutrophils and macrophages emerge at inflammatory sites as the first line of immune defense through CXC chemokines and proinflammatory cytokines.

The innate immune system plays a key role in protecting mucous membranes against various pathogens through pattern recognition receptors, including TLRs, which detect pathogens and function as signaling molecules [45]. Epipharyngeal innate immunity is triggered by the recognition of bacterial or viral nucleic acids, as well as products or other pathogen-associated molecular patterns by TLRs. Recognition of pathogens and endogenous TLR ligands promotes the activation of genes that encode proinflammatory cytokines, antimicrobial peptides, and other defense molecules [46]. Upregulation of TLRs has been observed in upper respiratory tract infections [47].

Following pathogen recognition via TLRs, dendritic cells in the mucosa produce proinflammatory cytokines that flow into the blood circulation, resulting in subsequent inflammation of distant organs, including the kidneys.

Abnormally upregulated levels of TLRs have been demonstrated in an animal model of IgAN [48] and patients with IgAN and IgA vasculitis (IgAV) [49,50,51,52,53]. Upregulation of TLR4 was found in blood mononuclear cells (PBMCs) from patients with IgAN [49,53], children with IgAN and IgAV [51], and children with IgAV [50,52]. The upregulation of TLR2, TLR 3, TLR5, and TLR9 [52,53] has also been reported in PBMCs of patients with IgAN and IgAV. Suzuki et al. investigated the expression levels of TLR2, TLR4, and TLR9 mRNAs in splenocytes from ddY mice, which spontaneously develop IgAN, and showed activation of the TLR9 pathway [48].

Therefore, it is assumed that residual hyperactivation of innate immunity may occur in some portions of MALT in patients with IgAN.

We have reported an extremely high incidence (682 out of 686 patients, 99.4%) of latent but significant epipharyngitis in patients with IgAN exhibiting hematuria, suggesting that chronic inflammation of the epipharynx may be a common background condition of IgAN [54]. It is conceivable that epipharyngitis, especially its chronic form, is the underlying condition responsible for the upregulation of TLRs in a considerable percentage of patients with IgAN.

### 3.3. Role of the Epipharynx–Kidney Axis in Glomerular Vasculitis

Regarding the underlying mechanism of synpharyngitic gross hematuria, we have proposed the concept of “epipharynx–kidney axis” [55]. Given that epipharyngeal T cells, as part of MALT, may behave similarly to tonsillar interfollicular T cells, namely, enhanced expression of CX3CR1 in CD8+T cells and other CXCR1+ cells, we postulate that upregulation of fractalkine/CX3CR1 interactions following activation of innate immunity related to acute epipharyngitis may play a crucial role in glomerular vasculitis, resulting in worsening of hematuria.

In response to foreign invaders, the activated lymphoid tissue of the epipharynx produces various inflammatory cytokines to clear pathogens and resolve pathogen-induced cell damage. Next, proinflammatory cytokines, including TNF-α, IFN-γ, and IL-1, flow into circulation from the epipharynx and enhance the expression of membrane-bound fractalkine in distant glomerular endothelial cells together with other adhesion molecules such as E- and P-selectins. This results in the recruitment of CX3CR1+ leukocytes and neutrophils into the glomeruli.

The accumulation of neutrophils in the glomeruli is essential in the development of *Glomerular vasculitis* in patients with IgAN [4]. Although the precise mechanism is unknown, we suspect that neutrophil accumulation mainly occurs locally and concomitantly with glomerular infiltration of CX3CR1+ cells [55].

Interleukin-17A (IL-17A), also called IL-17, is produced by the T helper 17 (Th17) subset of CD4+ T cells [56]. IL-17A is a proinflammatory cytokine that is uniquely positioned at the interface of innate and adaptive immunity. It induces the release of secondary proinflammatory cytokines in most epithelial, endothelial, and mesenchymal cells, leading to the recruitment and accumulation of neutrophils [57]. Importantly, CD8+ T cells, γδ T cells, and NKT cells, that is, CX3CR1+ leukocytes, also produce IL-17A [58,59]. Furthermore, increased serum IL-17 levels have been reported in patients with IgAN [60]. IL-17A can induce the production of proinflammatory cytokines, such as TNF and IL-1, from endothelial cells and infiltrating macrophages [61].

The increased concentration of local inflammatory cytokines, such as IL-1 and TNF-α, which are produced due to the fractalkine/CX3CR1 interaction, would induce glomerular endothelial expression of E- and P-selectins. Neutrophils are the major cells in the bloodstream and naturally express selectin ligands (P-selectin glycoprotein ligand-1: PSGL-1, E-selectin ligand-1: ESL-1, CD44, and Sialyl Lewis X: CD15) on their cell surface [62,63]. Therefore, the interaction of selectins in endothelial cells with selectin ligands in neutrophils occurs naturally, leading to the local accumulation of neutrophils in glomeruli.

Furthermore, neutrophils may change their phenotype (i.e., by increased expression and altered distribution of selectin ligands) in the inflammatory environment (high cytokine/chemokine) [64] of renal tissue and, thus, easily adhere to activated endothelial cells in the glomeruli. Consequently, neutrophils accumulate in the glomeruli and become activated through interactions with activated endothelial cells and the proinflammatory cytokine environment. This leads to the production and release of reactive oxygen species by proteolytic enzymes, leading to the rupture of the glomerular capillary walls, followed by the formation of crescentic lesions.

**Figure 9:**
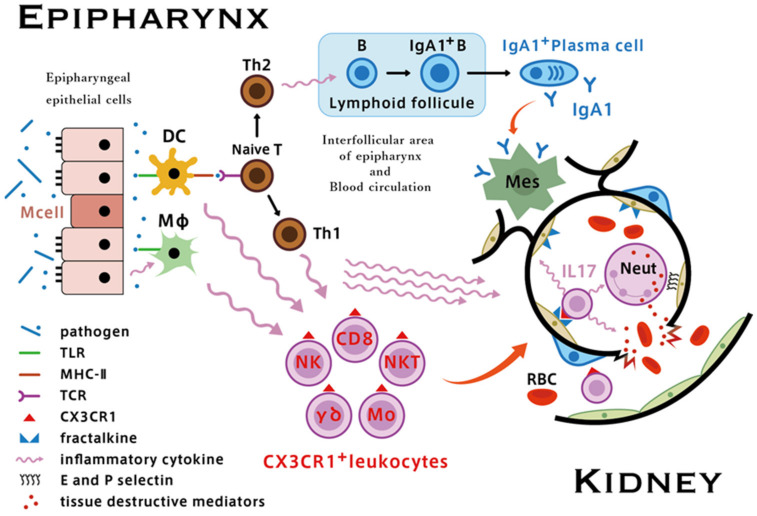
Putative mechanisms of the epipharynx–kidney axis causing glomerular vasculitis. In acute epipharyngitis, following pathogen recognition through TLRs, dendritic cells in the mucosa immediately produce proinflammatory cytokines. Simultaneously, infected epithelial cells and activated macrophages produce chemokines that upregulate the expression of CX3CR1 in monocytes, NK cells, CD8+ T cells, and γδ T cells. Circulating proinflammatory cytokines also enhance the expression of fractalkine and other adhesion molecules such as E- and P-selectins in glomerular endothelial cells. Upregulation of the fractalkine/CX3CR1 interaction and IL17 secreted by infiltrated glomerular CX3CR1+ leukocytes, which promotes the influx of neutrophils, leads to the development of *Glomerular vasculitis* and subsequent rupture of the glomerular capillary wall, which is clinically associated with worsening of hematuria [55].

As latent but significant epipharyngitis is present in most patients with IgAN, it is plausible that acute pharyngitis due to airway infection may contribute as a trigger for the epipharyngeal innate immune system, which is already upregulated in the chronically inflamed environment, leading to the upregulation of fractalkine/CX3CR1 interaction, resulting in the rupture of glomerular capillary walls, which is related to worsening hematuria (Figure 9).

### 3.4. Diagnosis of Epipharyngitis

A characteristic of acute or chronic epipharyngitis is easy bleeding due to submucosal congestion [39,65,66]. In acute epipharyngitis, discharge, petechial bleeding, and redness of the epipharyngeal surface are often observed. However, chronic epipharyngitis is sometimes difficult to diagnose using standard endoscopic procedures because the surface of the epipharyngeal wall is optically unremarkable in many patients. Therefore, chronic epipharyngitis can easily be overlooked during routine examinations by physicians.

**Figure 10:**
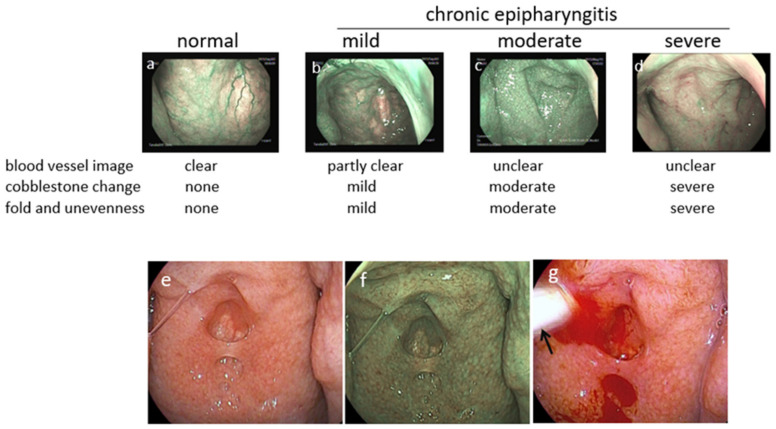
Endoscopic findings of chronic epipharyngitis. The vasculature is clearly visualized in the image-enhanced mode using band-limited light under normal conditions (**a**). In contrast, the vasculature is not visible due to submucosal edema and congestion, and the surface of the epipharynx becomes uneven and paved depending on the severity of chronic epipharyngitis (**b**–**d**). In a representative case of IgA nephropathy (**e**–**g**), the vasculature is obscure, and the surface exhibits a folded and cobblestone-like appearance (**e**,**f**). It is prone to bleeding by abrasion with a cotton swab (arrow) (**g**). Standard mode (**e**,**g**) and enhanced mode using band-limited light (**a**–**d**,**f**).

However, due to advances in image-enhanced endoscopic technology, band-limited light [67] has allowed physicians to identify chronic epipharyngitis by enhancing the congestion state, in contrast to the abnormal appearance of the vasculature on the surface of the epipharynx (Figure 10a–d). Furthermore, epipharyngitis can be easily diagnosed using epipharyngeal abrasive procedures, such as using a transnasal cotton swab with optimized bleeding during abrasion (Figure 10e–g) [65,66]. 

Nearly all patients with acute pharyngitis who suffer from throat pain, cough, and headache have severe epipharyngitis, regardless of concurrent kidney disease.

Notably, latent but significant epipharyngitis often exists even in healthy individuals. In our experience, chronic epipharyngitis was observed in 24 of 39 healthy control subjects (61.5%) despite the absence of pharyngitis-related symptoms [54]. This is consistent with a previous report showing moderate inflammation of the epipharynx in 69.8% of 202 Japanese schoolchildren [65].

### 3.5. Treatment of Chronic Epipharyngitis

For the treatment of chronic epipharyngitis, steroids, other immunosuppressive therapies, and antibiotics are not effective. Epipharyngeal abrasive therapy (EAT) is the only treatment known to be effective for chronic epipharyngitis. ZnCl_2_ solution is commonly used as a chemical solution for EAT in Japan. 

**Figure 11:**
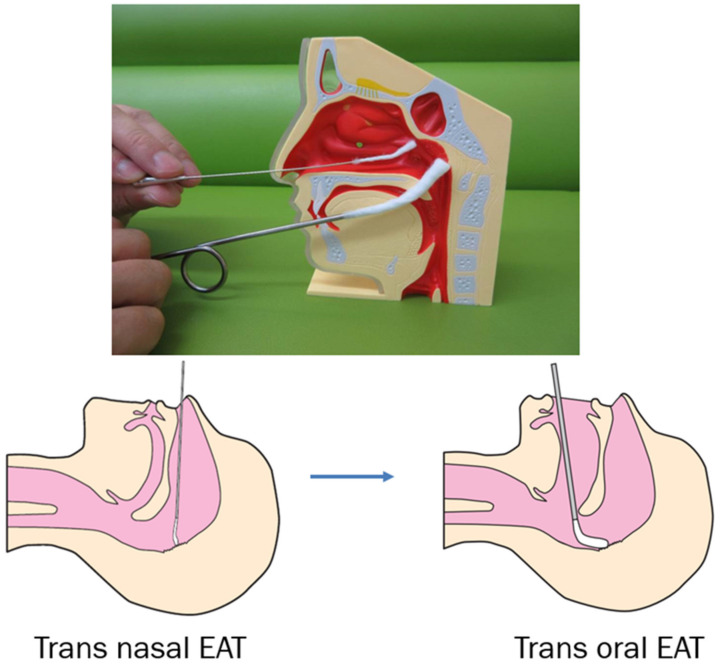
Schematic illustration of epipharyngeal abrasive therapy. A cotton swab soaked with 0.5% ZnCl_2_ solution was inserted through both nostrils and rubbed approximately 30 times on each side. Next, the entire epipharyngeal wall was scrubbed using a pharyngeal swab soaked in 0.5% ZnCl_2_ solution.

For this treatment, a sterile straight nasal cotton swab soaked with 0.5% ZnCl_2_ solution was inserted into one of the nostrils and pushed in a straight direction until resistance was felt. The swab was then repeatedly prodded in various directions to scrape the inflamed tissue. The procedure was repeated in the other nostrils. Next, the entire epipharyngeal wall was scrubbed using a pharyngeal swab soaked in 0.5% ZnCl_2_ solution (Figure 11).

### 3.6. Effects of EAT on Refractory IgAN and Potential IgAN

“Slowing the progression of nephropathy” is a universal treatment goal for IgAN. However, in Japan, where an annual urine screening system was developed and where IgAN is often detected at a relatively early stage, “remission and cure of IgAN” is a more acceptable treatment goal for patients [13]. To achieve clinical remission, it is essential to eliminate hematuria, which is an essential symptom of IgAN.

For this purpose, TSP therapy is widely used in Japan [68], and the elimination of hematuria is achieved in approximately 80% of cases with this treatment, regardless of histological grade [69]. In other words, remission of hematuria cannot be achieved with TSP therapy in the remaining 20% of patients with IgAN. Moreover, in some patients with IgAN, in whom the urinary occult blood completely disappears following TSP therapy, hematuria reappears after an acute episode of pharyngitis.

We performed EAT for patients with refractory IgAN on a trial basis. EAT was performed once a week in 24 patients with refractory IgAN [70]. The cohort study consisted of 19 patients with TSP-resistant IgAN and five with recurrent IgAN in whom hematuria remission was achieved in response to TSP therapy. The duration of EAT varied according to the improvement rate of epipharyngitis in each patient or their personal experiences (mean, 10.2 months; range, 1–30 months).

**Figure 12:**
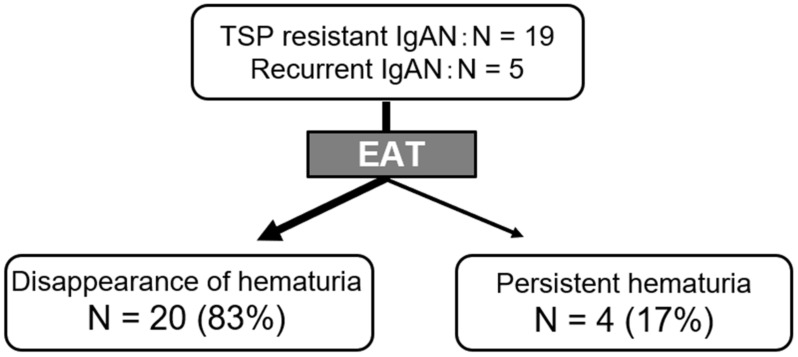
Effects of epipharyngeal abrasive therapy on hematuria in patients with IgAN with TSP resistant or relapsed course.

Hematuria remission was observed in 20 of 24 patients with IgAN (83.3%) following EAT. The duration between starting EAT and hematuria remission was 9.8 months on average (range, 1–26 months) (Figure 12).

In the early stages of IgAN, the degree of urinary protein is often low. Such patients do not become an indication for a renal biopsy until the urine protein increases, and it is common for it to be followed up without any treatment as “potential IgAN.”

To evaluate the efficacy of EAT on potential IgAN, we conducted a pilot cohort study of EAT in 54 potential patients with IgAN who had persistent glomerular hematuria for more than 3 months with minimum proteinuria and without hematuria-positive family members (to exclude patients with thin basement membrane disease or Alport syndrome) but did not undergo renal biopsy [71].

**Figure 13:**
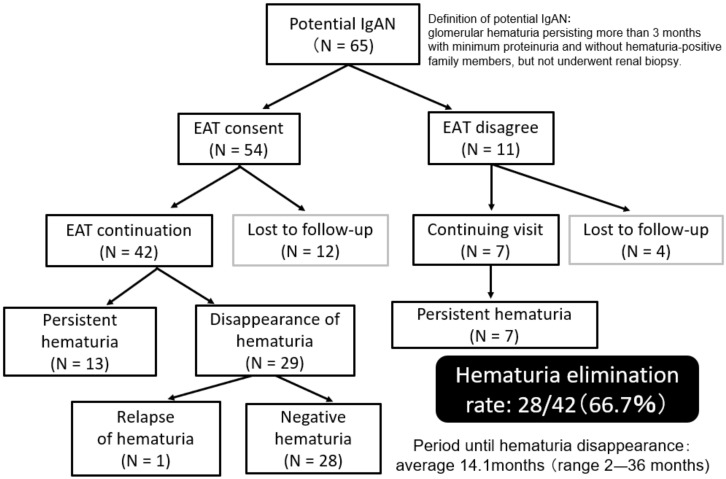
Effects of epipharyngeal abrasive therapy on hematuria in potential IgAN.

As 12 of 54 potential patients with IgAN withdrew from EAT and discontinued visits, the evaluation of EAT in hematuria was possible for the remaining 42 patients. Hematuria disappeared in 28 of 42 (66.7%) patients with potential IgAN. The duration between the start of EAT and the disappearance of hematuria was 14.1 months on average (range, 2–36 months). Meanwhile, the disappearance of hematuria was not observed in any of the seven potential patients who had been followed without EAT because they refused EAT (follow-up period > 24 months) (Figure 13).

The efficacy of EAT for IgAN cannot be concluded at present, but our preliminary cohort studies have suggested its usefulness. Therefore, it is expected that randomized controlled trials evaluating EAT in patients will be implemented in the future.

## 4. Treatment Options Based on the *Glomerular vasculitis* Related Conditions of IgAN

**Figure 14:**
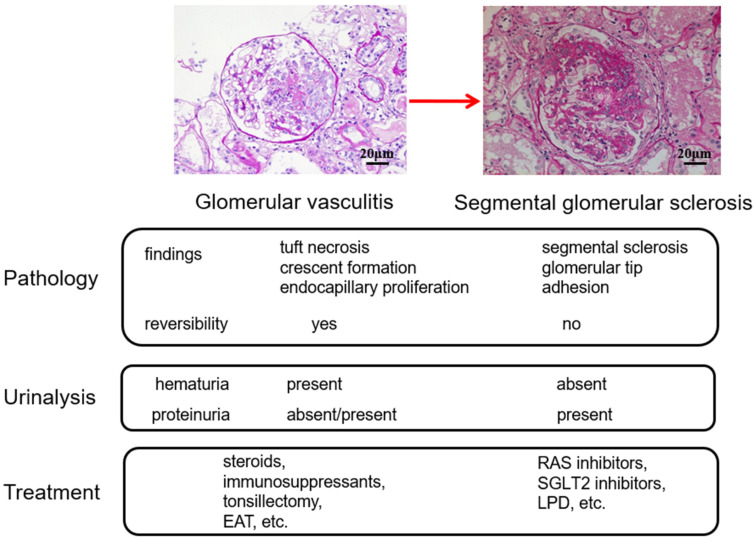
Treatment options based on the *Glomerular vasculitis* related pathologic conditions of IgAN. As *Glomerular vasculitis* and segmental glomerular sclerosis (SGS) differently coexist in IgAN, the key point of IgAN treatment is to estimate the element of *Glomerular vasculitis* and the element of the SGS from the findings of renal biopsy and urinalysis in each patient. It is also important to consider the limitation of histological assessment of *Glomerular vasculitis* by a limited amount of renal biopsy tissue. See the text for further details. RAS, renin-angiotensin system; SGLT2, sodium-glucose cotransporter 2; LPG, low-protein diet; EAT, epipharyngeal abrasive therapy.

Treatment options based on the clinicopathological conditions of *Glomerular vasculitis* are summarized in Figure 14. Focally and segmentally distributed smoldering *Glomerular vasculitis* and subsequent development of a healing scar lesion, called SGS, which eventually progresses to global glomerular sclerosis, is the major underlying mechanism responsible for the progression of IgAN. As the basic concept, it is also important to consider the limitation of histological assessment of glomerular vasculitis, whose lesion is focal and segmental in nature, by a limited amount of tissue obtained by renal biopsy.

Clinically, the magnitude of hematuria roughly represents the severity of glomerular vasculitis, while the severity of SGS lesions is reflected in the level of proteinuria. Unlike red blood cells, which are not reabsorbed by the urinary tubules, protein particles that leak from the glomeruli are reabsorbed by the proximal tubules; therefore, proteinuria is often absent in the early stages of IgAN.

From a therapeutic strategy perspective, treatments that lower glomerular pressure are effective in suppressing the progression of SGS lesions, and RAS inhibitors, low-protein diets, and possibly sodium-glucose cotransporter 2 (SGLT2) inhibitors [72,73] are the primary options. Meanwhile, treatment methods for ameliorating *Glomerular vasculitis* include steroids and immunosuppressants. In addition, tonsillectomy and EAT may be effective for *Glomerular vasculitis* because these treatments contribute to eliminating the initial hyperimmune response of innate and adaptive immunity in MALT.

## 5. Conclusions and Future Direction

Worsening hematuria, due to the aggravation of smoldering *Glomerular vasculitis*, accompanying pharyngitis is a distinctive clinical symptom of IgAN. However, the effect of epipharyngitis, which is an inevitable inflammation during acute pharyngitis, on hematuria or *Glomerular vasculitis* has not been fully elucidated. As significant chronic epipharyngitis is present in most patients with IgAN, chronic inflammation of the epipharynx may be a very common background condition of IgAN. Therefore, acute pharyngitis due to airway infection could trigger the epipharyngeal immune system, which is already upregulated in the chronically inflamed environment. This results in a hyperactivated innate immune system and upregulation of the fractalkine/CX3CR1 interaction.

Localized CX3CR1+ leukocyte inflammation in glomeruli with an increased concentration of inflammatory cytokines induced by fractalkine/CX3CR1 interactions leads to local accumulation and activation of neutrophils that produce and release tissue destructive mediators. As a result, glomerular vasculitis, the lesion responsible for the aggravation of hematuria, may occur.

Given that epipharyngitis and its effects on IgAN are not fully understood, we propose that the “epipharynx–kidney axis” may provide an important focus for future research. Furthermore, a treatment strategy aimed at ameliorating chronic epipharyngitis may prove worthwhile for patients with IgAN.

## Figures and Tables

**Table 1:** Phenotypic features of epipharyngeal lymphocytes.

	Epipharyngeal Lymphocytes				
	Healthy Control	Acute Pharyngitis ^a^	IgA Nephropathy	Tonsillar Lymphocytes	Peripheral Blood ^b^
	*n* = 9	*n* = 8	*n* = 32	*n* = 25	*n* = 7
CD4 (%)	29.1	35.2	41.7 **	39.2	42.2
CD8 (%)	8.4	9.9	12.1	9.3	29.8
CD4/CD8	4.28	4.12	4.25	4.63	1.48
CD20 (%)	57.8	54.6	43.6*	51.4	18.4
CD4+HLA-DR+/CD4+ (%)	30.5	53.3 ***	40.3 **^,#^	29.5	10.1
CD8+HLA-DR+/CD8+ (%)	41.6	66.4 **	58.9 **	45.6	23.0
CD19+CD69+/CD19+ (%)	41.4	30.1	29.1	46.9	0.8
CD19+CD5+/CD19+ (%)	4.1	10.5 **	12.8 **	20.0	22.4
CD56 (%)	0.6	1.0	1.2	1.5	15.4
CD57 (%)	1.3	0.5	2.2	5.3	14.2

* *p* < 0.05 vs. healthy control, ** *p* < 0.01 vs. healthy control, *** *p* < 0.001 vs. healthy control, ^#^ *p* < 0.01 vs. acute pharyngitis. ^a^ epipharyngeal lymphocytes were obtained from patients with acute pharyngitis without IgAN. ^b^ tonsils and peripheral blood samples were obtained from patients with IgAN. HLA-DR and CD69 are activation marker of T cell and B cell, respectively [44].

## Data Availability

Some of the data that support the findings of cited papers included in this review are available from the corresponding author upon reasonable request.
